# Update on liver transplantation-newer aspects

**DOI:** 10.3906/sag-2002-17

**Published:** 2020-11-03

**Authors:** Olga METİN, Cem ŞİMŞEK, Ahmet GÜRAKAR

**Affiliations:** 1 Department of Internal Medicine, Okmeydanı Training and Research Hospital, İstanbul Turkey; 2 Department of Gastroenterology, School of Medicine, Hacettepe University, Ankara Turkey; 3 Division of Gastroenterology and Hepatology, Johns Hopkins University School of Medicine Liver Transplant Program Baltimore, Maryland USA

**Keywords:** Extended criteria donor, liver transplantation, marginal liver grafts, severe alcoholic hepatitis

## Abstract

Liver transplantation (LT) remains the only therapeutic option offering gold standard treatment for end-stage liver disease (ESLD) and acute liver failure (ALF), as well as for certain early-stage liver tumors. Currently, the greatest challenge facing LT is the simple fact that there are not enough adequate livers for all the potential patients that could benefit from LT. Despite efforts to expand the donor pool to include living and deceased donors, organ shortage is still a major problem in many countries. To solve this problem, the use of marginal liver grafts has become an inevitable choice. Although the definition of marginal grafts or criteria for expanded donor selection has not been clarified yet, they are usually defined as grafts that may potentially cause primary nonfunction, impaired function, or late loss of function. These include steatotic livers, older donors, donors with positive viral serology, split livers, and donation after cardiac death (DCD). Therefore, to get the best outcome from these liver grafts, donor-recipient selection should be vigilant. Alcohol-related liver disease (ALD) is one of the most common indications for LT in Europe and North America. Traditionally, LT for alcoholic liver disease was kept limited for patients who have achieved 6 months of abstinence, in part due to social and ethical concerns regarding the use of a limited resource. However, the majority of patients with severe alcoholic hepatitis who fail medical therapy will not live long enough to meet this requirement. Besides, the initial results of early liver transplantation (ELT) without waiting for 6 months of abstinence period are satisfactory in severe alcoholic hepatitis (SAH). It will be important to take care of these patients from a newer perspective.

## 1. Introduction

Since the first procedure performed by Thomas E. Starzl in the 1960s, liver transplantation (LT) has become the gold standard for the treatment of end-stage liver disease (ESLD), acute liver failure, and some selected liver tumors [1]. Despite the efforts to increase to donor pool by increasing the usage of live and deceased donors, there has been an unmet need for donor livers in the United States (US) and universally [2,3]. The demand for liver has been steadily expanding. Only in the US, annually, about 11,000 patients with ESLD get enlisted, while annual liver transplantations are in the range of 6000–7000 [2]. To overcome the organ shortage problem, transplantation centers had to expand their criteria for donor selection. With the expansion of donor suitability criteria, the use of marginal grafts has become mandatory. Marginal grafts or expanded donors are grafts that may potentially cause primary nonfunction, impaired function, or late loss of function, although there is not a clear-cut definition [3,4].

In this review we defined marginal grafts as grafts that carry potential risks of early or late loss of function, meaning older donors, donors with steatosis, hepatitis, human immunodeficiency virus (HIV) or split liver, or donors after cardiac death.

As post-LT survival rates have been steadily improving, the mean age of donors and recipients increased with a resulting increase in the use of marginal donors. Improvements in surgical techniques, advances in postoperative care, and developments in new immunosuppressive medications have also contributed to this. Recent United Network for Organ Sharing (UNOS) data shows that 1-year post-LT survival is around 85%–90% and 10-year survival is around 50%. 

Before 2002, prioritization of liver transplantation was performed according to the Child-Turcotte-Pugh score. This system was based on the presence of subjective criteria such as ascites and encephalopathy to predict short term mortality risk. To overcome this hurdle, a more objective alternative was the Model for End-Stage Liver Disease (MELD) score. After the implementation of MELD, wait-list mortality has dramatically declined [5–7]. Besides this score, the presence of fulminant hepatic failure, metabolic liver disease, or complications from chronic liver disease such as variceal bleeding and development of hepatocellular cancer, are also considerable factors to proceed with transplantation.

Besides the criteria above, there are some diseases in which the MELD score is not directly correlated with survival. These “MELD-exceptions” are hepatocellular cancer, hepatopulmonary syndrome (HPS), portopulmonary hypertension (PPH), familial amyloid polyneuropathy, cystic fibrosis, or cholangiocarcinoma after chemoradiotherapy protocol. Other considerations, such as donor age (D-MELD) and frequent cholangitis episodes in patients with primary sclerosing cholangitis have been emerging as important factors that predict prognosis, but they are not MELD-exception points by consensus yet [8,9]. Recently, serum sodium has also been included in MELD calculations and used as MELD-Na (especially in patients with low serum sodium) in the US [10].

After the final decision on LT, screening and evaluation of possible comorbidities is of crucial importance for patients on the transplant waitlist. Even though postLT survival rates have increased with recent developments in surgical techniques and medical care, liver recipients still have lower short-term survival compared to the age-sex matched general population [11]. Most frequent complications are due to cardiovascular diseases in the long-term follow up of liver transplant recipients. Cardiovascular events comprise almost 19%–42% of mortalities in this group of patients [12,13]. Mortality rates increase in ESLD patients who had coronary artery disease by angiography in the preLT period [14]. Thus, a preLT evaluation protocol should be able to detect underlying cardiovascular disease. Single positron tomography, myocardial perfusion scintigraphy, and dobutamine stress echocardiography are valuable to evaluate coronary artery disease. Coronary calcium score (CCS) calculated by computerized tomography is known to be correlated with the severity of coronary artery disease and can predict the cardiovascular risk in ESLD patients [15,16].

## 2. Donor age

Organ shortage in liver transplantation will potentially lead to an increased usage of older donors in the future [17]. In the US, donors aged more than 50, comprise 33% of donors, while in some European countries this ratio increases to greater than 50% [18]. The primary problems with older age donor grafts are impaired regeneration capacity after transplantation and being prone to ischemic and reperfusion injury. These grafts are more vulnerable to hepatitis C (HCV) recurrence and graft fibrosis and cirrhosis develop faster [19,20]. Fortunately, the synthetic capacity of liver is similar in older grafts due to the dual blood supply [21].

The definition of an older donor shows variability among different transplantation centers. Age threshold can change between deceased donor liver transplantation (DDLT) and living donor liver transplantations (LDLT). Most studies have defined the age threshold between 65–70 years in DDLT, while 50–60 years in LDLT [22,23]. One study calculated liver volumes after LDLT on postoperative 7th day and 3–6 months and compared them with donor age <30 years versus donor age >50. In patients with donor age >50, the regenerative capacity of the liver decreased with age as an independent risk factor [19]. In the case of LDLT, the condition of the recipient is not the sole problem, as the donors’ survival and complication rates after hepatectomy are at least of equal importance. Impaired regeneration capacity increases morbidity for both the donor and the recipient. The regeneration problem is not important in DDLT as the whole liver is used as a graft [24]. Previous studies have shown that in LDLT donor age >50 or 60 resulted in lower patient and graft survival rates if the recipients were older, HCV positive, and their MELD score was greater than 20 [20,23–26]. Postoperative complication rates and severity were found to be similar between donors <50 years of age and donors >50 years of age [26,27].

A few studies showed that in HCV positive recipients graft loss and recurrent HCV infection followed by hepatic fibrosis and development of cirrhosis were faster when donors were older. A recent consensus held in Paris recommended not to use older grafts in HCV positive recipients [22,28,29]. However, at the same time, a cure of the HCV infection is possible with direct-acting antiviral drugs. The treatment of HCV infection in live donors before or after transplantation might enable us to use older donor grafts in HCV positive recipients. In a short time, older grafts used in LDLT will potentially result in better patient and donor survival rates if used in HCV negative recipients with low MELD scores if they do not have steatosis or increased ischemia time due to technical reasons.

## 3. Liver graft steatosis

Hepatic steatosis has 2 subgroups: macro- and microsteatosis. Microvesicular steatosis is not associated with poor prognosis after transplantation; in contrast, macrovesicular steatosis is associated with primary or early weak donor function [30]. Why does macrovesicular steatosis lead to poor graft function? Its pathogenesis is not exactly clear. However, macrovesicular steatosis leads to impaired hepatic microcirculation, which makes liver more susceptible to cold ischemia and ischemia reperfusion injury [30]. Grafts with lower than 30% steatosis are not associated with worse posttransplant prognosis. On the other hand, grafts with 30%–60% steatosis are preferable when the donor has normal liver functions, the donor is under 60 years of age, cold ischemia time is below 8 h, with good graft removal conditions in recipients who meet the following criteria: HCV negative with a MELD score lower than 20. In the cases of recipients or donors with greater than 30% steatosis, the transplant team should consider the above risks [31].

Liver steatosis is a more important topic in living LDLT than DDLT since it increases both the donor and the recipient morbidity due to poorer graft functions [31,32]. Previous studies showed no relationship between primary or early poor graft functions and steatosis up to 30% [33–35]. In mild steatosis up to 60%, both graft and recipient survival rates with nonsteatotic grafts were observed if graft volume was higher than 40% of standard liver volume. Severe liver steatosis affects both graft and recipient survival rates [36]. Biliary complications were seen more often in these grafts in the first 3 months after transplantation. Besides, survival rates of grafts with severe steatosis (>60%) were significantly shorter and approximately 25% in 1 year [37]. Similarly, a recent metaanalysis reported that grafts with moderate to severe steatosis showed lower survival rates compared to grafts with no steatosis or mild steatosis. Macrovesicular steatosis also increases the probability of poor graft functions (PNF) [30].

Unlike cadaveric liver transplantation, steatosis in living donors may be reversible. A short-term intense protein-rich diet, exercise, drugs like fibrates and omega-3 fatty acids may reduce liver steatosis. In some studies, these methods reduced steatosis successfully in donors and improved the postoperative outcomes of donors and recipients [38,39].

## 4. Obesity

Obesity is on the rise around the world and is threatening the liver donor pool. According to 2012 data, 69% of the entire population of the US was overweight (body mass index [BMI] >25) and 35% was obese (BMI > 30) [40]. Obesity is a known strong risk factor for liver steatosis. In a study, 76% of BMI > 28 living donors had steatosis in liver biopsies [41].

Graft steatosis is associated with worse outcomes in recipients after liver transplantation. These include ischemia reperfusion injury, biliary strictures, primary graft failure, and lower survival rates in 1 year [42,43].

Although negative effects of graft steatosis are well-known effects of obesity alone without steatosis on liver transplantation are controversial. Recent studies reported that in select obese donor groups (BMI ≥ 30 but ≤ 35) of nonsteatotic livers and without accompanying cardiovascular comorbidities including hypertension, diabetes mellitus, and dyslipidemia, donor hepatectomy may be feasible. Both recipients of donors with BMI > 30 and donors had similar outcomes with donors with BMI < 30 in the short and long-term. Obesity is also a risk factor for postoperative complications. These complications include pulmonary infections, delayed wound healing and wound infection, and thrombotic events [44,45]. The length of hospital stay is longer in obese patients and the cost of treatment is higher [46].

Knak et al. have observed that people with obesity without liver steatosis and cardiovascular comorbidities may safely become donors [47]. Dindo et al. evaluated the elective surgical results of 6336 patients and reported that 26% were obese. They concluded that obesity was not a risk factor for postoperative complication rate [48].

## 5. Chronic hepatitis of grafts

Both donor and recipient infection with hepatitis viruses affect posttransplant outcomes. Previously, HBV or HCV positivity in grafts was an exclusion criterion for transplantation. Along with prophylaxis against hepatitis B virus (HBV) via the development of HBV vaccine, use of Hepatitis B immunoglobulin, and use of nucleoside analogs led to the use of hepatitis B surface antigen (HBsAg) and hepatitis B core antigen (HBcAg) positive grafts in liver transplants [49]. Similarly, the development of direct-acting antiviral agents (DAA) against HCV led to transplantation of HCV infected liver grafts to both HCV positive and negative recipients [50].

Grafts in antiHBcAg antibody (HBcAb) positive donors carry the risk of HBV transmission and most of these donors have occult HBV infection [51]. After transplantation of HBcAb-positive grafts, the risk of de novo HBV (DNHB) infection in HBV-naïve recipients is 58% higher; a lower risk is observed with previous HBV vaccination or HBV infection (HBsAb+, HBcAb+) [52]. Previous grafts with HBcAb-positive donors were used in HBV-naïve recipient; DNHB risk was found to be lower in these recipients with the use of lamivudine [52].

In light of these data, the American Society of Transplantation (AST) consensus guideline recommends long term treatment of HBV-naïve recipients with HBcAb-positive donors with nucleoside analogs for prophylactic purposes [53].

Due to the rapid transmission and progression of HBV infection in grafts, and because of the loss of 50% of grafts in 2 years, chronic HBV was a definite contraindication for liver transplantation in the 1980’s [54,55]. Hepatitis B immunoglobulin and following antiviral treatment led to dramatic results in clinical outcomes. Nowadays, posttransplant chronic HBV patients have better outcomes than other transplantation indications [56,57]. Nevertheless, HbsAg-positive grafts without delta hepatitis and without histologic signs of liver disease are considered for liver transplantation [58]. Any HBV infected patient should take antiviral treatment. Posttransplant HBIG administration is a common practice in transplant centers depending on recipients’ risk status to keep HBsAb titers between 100–500 IU/mL [53].

In the aspect of HCV, HCV-positive grafts were previously only transplantable to HCV-positive recipients [59,60]. Due to the potential posttransplant transmission of HCV to the recipient and the course of HCV in untreated patients, transplantation of HCV positive grafts is still uncommon [61]. Direct-acting antiviral agents (DAA) are both very effective in treatment and well-tolerated in patients with HCV infection. DAA has success rates above 95% [62]. HCV-positive grafts have similar graft and recipient survival rates in HCV-negative recipients if periportal fibrosis (F2 Ishak) is absent during the pretransplant period [63]. HCV viremia or de novo HCV infection is detectable by the positivity of HCV RNA in serum. The mean time for positivity is 1 week after transplantation [53].

Recurrent HCV infection warrants prompt treatment. Laboratory, clinical, or histologic findings should not cause a delay. The choice of DAA depends on the patients’ immunosuppressive regimen and potential drug interactions. AST guidelines recommend starting a pan-genotypic agent in the early posttransplant period without delay for genotype analysis [53].

## 6. Human immunodeficiency virus

The worldwide prevalence of human immunodeficiency virus (HIV) has reached 37 million [64]. With the development of antiretroviral treatment, HIV-infected patients have reached a normal lifetime, and HIV-unrelated causes have become major determinants for their survival [64]. Liver disease is one of the leading causes of death unrelated to AIDS, reaching 10% [64]. An important reason for this increased prevalence is a concomitant infection of HBV and HCV with HIV, reported as more than 10% and 30%, respectively [64]. Thus, the promotion of organ transplantation in this population is of great importance, since the survival rates of HIV-infected recipients are comparable to noninfected recipients, albeit with 3 times higher acute rejection rates. 

The first efforts to transplant HIV-positive organs were hampered by the poor outcomes in the 1980’s, resulting in strict prohibitions in many countries. A decade later, following the advent of antiretroviral therapies, a transplant from HIV patients is deemed feasible. Particularly in countries with high HIV prevalence, liver transplantation from HIV-positive donors has become an appealing option. Moreover, nearly two-thirds of HIV-positive patients are willing to donate their organs to HIV-positive recipients. They have unique motivations such as overcoming HIV-related stigmas and empathy for other infected patients [65].

Muller from the South African Republic spearheaded HIV-positive organ transplantation. In his pioneering series, 27 HIV-infected patients had kidney transplantation from HIV-infected donors. In these patients, 3- and 5-year graft and recipient survival rates were found to be similar to non-HIV infected counterparts. After similar reports from the United Kingdom and Switzerland [66], HIV Organ Policy Equity (HOPE) Act passed in the US in 2013. With this law, the use of HIV-positive organs as grafts has begun in the US. In March 2016, the first liver transplantation of an HIV-positive recipient from an HIV-positive donor was performed at Johns Hopkins University [67]. For now, HIV-positive organs are transplantable only to HIV-positive patients. First-time transplantation of an HIV-positive organ to an HIV-negative recipient in the world was in the South African Republic in 2017. In this case, an HIV-positive mother donated her liver to her baby with biliary atresia. A special ethics committee decision and legal permissions were followed by standard transplant surgery. Before the surgery, the mother had antiretroviral treatment and the baby had preoperative prophylaxis. One year after transplantation, both the baby and the mother were both in good condition. With this transplantation, the probability of usage of HIV-positive organs in HIV-negative patients is considerable [68], albeit long-term outcomes remain unclear.

A recent study explored another benefit of the HOPE act. Every organ is prescreened for HIV antibody and nucleic acids before pursuing transplantation, however, these tests are known to have nonnegligible false-positive rates. Before the act, these organs were unusable if either one was positive, but with the act, the organs are transplantable to seropositive recipients. The estimated number for this organ pool is 50–100 per year in the US [69].

## 7. Donor after cardiac death

Since the 1990’s, organs of donors after brain death (DBD) have been used in many transplantation centers. Organs of donors after cardiac death (DCD) comprise 5% of all cadaveric donors [70,71]. Notably, these organ donation procedures have started right after the determination of death by cardiorespiratory criteria. The quality of the donor is the most important factor determining peri- and posttransplantation organ functions. A metaanalysis consisting of 25 studies evaluated the outcomes of 62,000 liver transplantation recipients. 

Ischemic type biliary strictures were commonly observed in livers from DCD with a reduced total graft and recipient survival [72]. Although the mechanism of ischemic cholangiopathy is unclear, possible mechanisms are longer duration of hot ischemia causing blood stasis and clots in peribiliary microcirculation [73,74].

## 8. Split liver grafts

Split liver transplantation (SLT) is the sharing of a liver of an adult cadaver donor between an adult and a child recipient. SLT has become an option to increase the donor pool in child patients. After SLT, complications such as biliary leaks, biliary strictures, and hepatic artery thrombosis are more common in adult recipients than in children in 10 years [75,76]. These complications are less frequent in further years [77,78]. A successful SLT depends on 3 factors, including careful recipient and graft selection, reducing risk factors associated with bad results, and trying to keep cold ischemia time as short as possible during liver splitting [79].

## 9. Severe alcoholic hepatitis

Alcohol-related liver disease (ALD) is the most common indication of liver transplantation in Europe and the US [80]. Severe alcoholic hepatitis (SAH) is the presence of jaundice and hepatic decompensation in individuals who consume excessive alcohol [81]. Short term mortality of these patients is high, and 6-month mortality is less than 30% [82,83]. Corticosteroid treatment is useful if not contraindicated and some patient groups do not respond to steroid treatment. For these patients, liver transplantation is the only option [84]. Traditionally, liver transplantation in ALD patients awaits 6 months of alcohol cessation due to limited donor pool as well as social and ethical concerns [81]. Unfortunately, most of these patients cannot survive even 6 months to complete this abstinence period [81]. Hence, early liver transplantation is considered for severe alcoholic hepatitis. The first study by Mathurin et al. was from Europe and included 6 centers from France and 1 center from Belgium between 2005 and 2010. In this study, 26 patients had liver transplantation and survival rates have increased significantly at 6 months and 2 years [83]. Following this article in 2011, 9 patients with SAH after liver transplantation in the US from Mount Sinai hospital had similar results [82]. Six-month survival rates were as high as 89% in transplanted patients [82]. A larger study from Johns Hopkins including 46 carefully selected SAH patients who had undergone LT, had similar 1-year outcomes (97% patient survival and 93% graft survival in the SAH group) and recidivism (28% in the SAH group) when compared to 34 patients with more than 6 months of sobriety [84].

 All these studies showed that early liver transplantation in selected patients with SAH who fail to respond to medical treatment may benefit from liver transplantation with a 6-month survival rate of 77% and 100% [82,84].

The American Consortium of Early Liver Transplantation for Alcoholic Hepatitis (ACCELERATE-AH) group evaluated the results of early liver transplantation of 147 patients with SAH without waiting for 6 months of alcohol abstinence period in 12 transplantation centers and reported a 1-year survival rate of 94%, and 3-year survival rate of 84% (85). Data from Europe and the US confirm the need for reconsideration of the rule of 6 months of alcohol abstinence period [80].

Alcohol consumption after liver transplantation is a major problem for both ALD and SAH. Studies showed similar rates of reuse of alcohol for early liver transplantation compared to late transplantation after 6 months of abstinence period. In a prospective study conducted by Di Martini et al. on 167 patients with transplantation after 6 months of alcohol abstinence, the alcohol recidivism rates were 21% and 32% in 1 year and 3 years, respectively [86].

In the American Consortium study by Lee et al. [85], alcohol recidivism in 147 SAH patients with early LT was 25% and 34% in 1 and 3 years, respectively. Studies showed similar rates of alcohol relapse in early liver transplantation (transplantation in 6 months) and transplantation after 6 months of alcohol abstinence period [87]. These studies support the reconsideration of 6 months of alcohol abstinence period in ALD. Patients with SAH who failed to respond to medical treatment have a survival rate of almost above 80% after liver transplantation. But reuse of alcohol increases morbidity and graft loss, especially in heavy drinkers. The major problem is still the reuse of alcohol in these patients. 

## 10. Conclusion

In this review, we aimed to discuss the requirement for marginal liver grafts caused by a limited donor pool and the increasing need for liver transplantation. Marginal grafts are associated with poor graft outcomes. In light of the data, careful patient and graft selection may contribute to better outcomes. The graft pool is insufficient, and demand is rapidly increasing. Marginal grafts still seem to be the only option to increase the donor pool. Besides, the results of early liver transplantation without waiting for 6 months of abstinence period are encouraging in SAH. Care of such patients needs a newer perspective, especially for selected groups.

## References

[ref1] (u2e72). Homotransplantation of the liver in humans. Surgery, Gynecology & Obstetrics.

[ref2] u2e74 et al. OPTN/SRTR 2017 Annual Data Report: Liver. u2e6d 2019 (Suppl.

[ref3] (2008). The marginal liver donor-an update. Transplant International.

[ref4] (2010). Outcome of liver transplantation based on donor graft quality and recipient status. Transplantation Proceedings.

[ref5] (2000). Edwards EB. Liver Transplantation.

[ref6] (2002). The new liver allocation system: moving toward evidence-based transplantation policy. Liver Transplantation.

[ref7] (u2e6e). Indications for Liver Transplantation.

[ref8] (2009). D-MELD, a simple predictor of post liver transplant mortality for optimization of donor/recipient matching. American Journal of Transplantation.

[ref9] (2011). A revised model for end-stage liver disease optimizes prediction of mortality among patients awaiting liver transplantation. Gastroenterology.

[ref10] (2001). A model to predict survival in patients with end-stage liver disease.

[ref11] (u2e6c). Differences in long term survival among liver transplant recipients and the general population: a population-based Nordic study. Hepatology.

[ref12] (2010). Evolution of causes and risk factors for mortality post-liver transplant: results of the NIDDK long-term follow-up study. American Journal of Transplantation.

[ref13] (u2e69). u2e6a et al. Long-term outcomes in patients undergoing liver transplantation for nonalcoholic steatohepatitis-related cirrhosis. Transplantation.

[ref14] (2010). Multi-vessel coronary artery disease predicts mortality, length of stay, and pressor requirements after liver transplantation. Liver Transplantation.

[ref15] (2004). Coronary artery calcium score combined with Framingham score for risk prediction in asymptomatic individuals. JAMA.

[ref16] (u2e68). Comparison ofcalcium scores from thick- and thin-image slice-computed tomography scanning in predicting future coronary events. American Journal of Cardiology.

[ref17] (2015). Reappraisal of upper age limit for adult living-donor liver transplantation using right lobe grafts: an outcome analysis. European Journal of Gastroenterology & Hepatology.

[ref18] (2016). How important is donor age in liver transplantation? u2e76ogy.

[ref19] (2012). Donor age affects liver regeneration during early period in the graft liver and late period in the remnant liver after living donor liver transplantation. World Journal of Surgery.

[ref20] (u2e5e). Outcomes of livingdonor liver transplantation using elderly donors. Annals of Surgical Treatment Research.

[ref21] (2006). Ageing and the liver. Acta Gastroenterologica Belgica.

[ref22] (2018). Use of elderly donors in liver transplantation: A paired-match analysis at a single center. Annals of Surgery.

[ref23] (u2e5d). Impact of elderly donors for liver transplantation: A single-center experience. Liver Transplantation.

[ref24] (u2e5c). Effect of donor–recipient age matching in living donor liver transplantation. Transplant Proceedings.

[ref25] (u2e5a). Liver transplantation in Japan: registry by the Japanese Liver Transplantation Society. Hepatology Research.

[ref26] (u2e58). Impact of donor age and recipient status on left-lobe graft for living donor adult liver transplantation. Transplant International.

[ref27] (u2e57). Utilization of elderly donors in living donor liver transplantation:when more is less? Liver Transplantation.

[ref28] (u2e54). Recipient age affects long-term outcome and hepatitis C recurrence in old donor livers following transplantation. Liver Transplantation.

[ref29] (u2e52). u2e53 et al. Liver Transplantation.

[ref30] (2015). Donor hepatic steatosis and outcome after liver transplantation: a systematic review. Journal of Gastrointestinal Surgery.

[ref31] (2007). Hepatic steatosis is a risk factor for postoperative complications after major hepatectomy: a matched case-control study. Annals of Surgery.

[ref32] (u2e51). Liver steatosis assessed by preoperative MRI: an independent risk factor for severe complications after major hepatic resection. Surgery.

[ref33] (u2e4f). Effect of pure microsteatosis on transplant outcomes after living donor liver transplantation: a matched case-control study. Liver Transplantation.

[ref34] (u2e4e). Liver regeneration and function in donor and recipient after right lobe adult to adult living donor liver transplantation. Transplantation.

[ref35] (u2e49). u2e4a et al. Transplantation.

[ref36] (u2e48). u2e4b et al. Transplantation Proceedings.

[ref37] (u2e46). Grade of deceased donor liver macrovesicular steatosis impacts graft and recipient outcomes more than the donor risk index. Journal of Gastroenterology & Hepatology.

[ref38] (u2e44). Short-term intensive treatment for donors with hepatic steatosis in living-donor liver transplantation. Transplantation.

[ref39] (2011). Omega-3 fatty acids reduce hepatic steatosis and consequently attenuate ischemia reperfusion injury following partial hepatectomy in rats. Digestive and Liver Disease.

[ref40] (2011). Prevalence of childhood and adult obesity in the United States,.

[ref41] (u2e43). Body mass index as a predictor of hepatic steatosis in living liver donors. Liver Transplantation.

[ref42] (2015). Donor Hepatic Steatosis and Outcome After Liver Transplantation: a Systematic Review. Journal of Gastrointestinal Surgery.

[ref43] (2014). Liver-specific deceased donor risk indices. Hepatology Research.

[ref44] (2016). Complications of bariatric surgery–What the general surgeon needs to know. Surgeon.

[ref45] (2011). Perioperative pulmonary embolism: Diagnosis and anesthetic management. Journal of Clinical Anesthesia.

[ref46] (2015). da Silva EN. PLoS ONE.

[ref47] (u2e40). a Contraindication for Live Liver Donation. Donor BMI > 30.

[ref48] (2003). Obesity in general elective surgery. Lancet.

[ref49] (2016). Liver grafts from hepatitis B surface antigen-positive donors: a review of the literature. World Journal of Gastroenterology.

[ref50] (u2e3f). u2e42 et al. American Journal of Transplantation.

[ref51] (2010). Liver grafts from anti-hepatitis B core positive donors:a systematic review. Journal of Hepatology.

[ref52] (2011). Risk of de novo hepatitis in liver recipients from hepatitis-B core antibody-positive grafts–a systematic analysis. Clinical Transplantation.

[ref53] (2019). Viral hepatitis: Guidelines by the American Society of Transplantation Infectious Disease Community of Practice. Clinical Transplantation.

[ref54] (u2e3c). Hepatitis B virus reinfection after orthotopic liver transplantation. Serological and clinical implications. Journal of Hepatology.

[ref55] (1992). Rosenthal S et al. Gut.

[ref56] (u2e3e). Liver transplantation for HBV‐ related cirrhosis in Europe: an ELTR study on evolution and outcomes. Journal of Hepatology.

[ref57] (u2e4d). u2e35 et al. Long‐term trends in chronic hepa‐ titis B virus infection associated liver transplantation outcomes in the United States. Journal of Viral Hepatitis.

[ref58] (u2e36). u2e34 et al. Long-term follow-up and outcome of liver transplantation from hepatitis B surface antigen positive donors. World Journal of Gastroenterology.

[ref59] (2010). Liver allografts from hepatitis C positive donors can offer good outcomes in hepatitis C positive recipients: a US National Transplant Registry analysis. Transplant International.

[ref60] (2017). Changes in Utilization and Discard of Hepatitis C-Infected Donor Livers in the Recent Era. American Journal of Transplantation.

[ref61] (2012). Risk of advanced fibrosis with grafts from hepatitis C antibody-positive donors: a multicenter cohort study. Liver Transplantation.

[ref62] (2014). An interferon-free antiviral regimen for HCV after liver transplantation. The New England Journal of Medicine.

[ref63] (2016). Liver retransplantation for recurrence of HCV-related cirrhosis using Hepatitis C-positive allografts:a 19-Year OPTN analysis. Annals Transplantation.

[ref64] (2019). Solid Organ Transplantation in HIV-Infected Recipients: History, Progress, and. Current HIV/AIDS Reports.

[ref65] Perceptions, motivations, and concerns about living organ donation among people living with HIV.

[ref66] (2017). The Times They are a-Changing: HOPE for HIV to HIV Organ Transplantation. Transplantation.

[ref67] (2016). the ethic of organ transplantation from HIV-positive donors. Annals of Internal Medicine.

[ref68] (u2e2c). Living donor liver transplant from an HIV-positive mother to her HIV-negative child: opening up new therapeutic options. AIDS.

[ref69] Organs from deceased donors with false‐positive HIV screening tests: An unexpected benefit of the HOPE act.

[ref70] (2014). Organ donation and transplantation in the UK—the last decade: a report from the UK national transplant registry. Transplantation.

[ref71] (u2e30). u2e29 et al. OPTN/SRTR 2015Annual data report: liver. American Journal of Transplantation.

[ref72] (2014). A meta-analysis and meta-regression of outcomes including biliary complications in donation after cardiac death liver transplantation. Transplant International.

[ref73] (u2e2a). Comparison of long-term outcomes and quality of life in recipients of donation after cardiac death liver grafts with a propensity-matched cohort. Liver Transplantation.

[ref74] (u2e28). u2e26 et al. American Journal of Transplantation.

[ref75] (u2e39). Split liver transplantation using hemiliver graft in the MELD era:a single center experience in the United States. American Journal of Transplantation.

[ref76] (u2e23). The incidence, timing, and management of biliary tract complications after orthotopic liver transplantation. Annals of Surgery.

[ref77] (u2e24). Outcomes with split liver transplantation are equivalent to those with whole organ transplantation. Journal of the American College of Surgeons.

[ref78] (2017). Intention to split policy: a successful strategy in a combined pediatric and adult liver transplant center. Annals of Surgery.

[ref79] (2017). Split liver grafts can benefit both pediatric and adult liver transplant recipients and programs. Pediatric Transplantation.

[ref80] (2020). Early Liver Transplantation in Acute Alcoholic Hepatitis.

[ref81] (u2e19). The Role of Liver Transplantation in Alcoholic Hepatitis.

[ref82] (u2e17). Early liver transplantation for severe alcoholic hepatitis in the United States–a single-center experience. American Journal of Transplantation.

[ref83] (u2e15). Early liver transplantation for severe alcoholic hepatitis. New England Journal of Medicine.

[ref84] (u2e14). Liver transplantation for severe alcoholic hepatitis, updated lessons from the world’s largest series. Journal of the American College of Surgeons.

[ref85] (u2e11). Outcomes of early liver transplantation for patients with severe alcoholic hepatitis. Gastroenterology.

[ref86] (u2e10). Alcohol consumption patterns and predictors of use following liver transplantation for alcoholic liver disease. Liver Transplantation.

[ref87] (2019). Pro: Liver Transplantation Should Be Considered in Select Patients With Acute Alcoholic Hepatitis.

